# Phenylalanine coordinates antioxidant responses and ion homeostasis to enhance salinity tolerance in maize

**DOI:** 10.1080/15592324.2026.2709225

**Published:** 2026-07-30

**Authors:** Sidra Ghafoor, Muhammad Ahmad, Nazia Ishfaq, Kaleem Ul Din, Areeba Abdul Khaliq, Hossam S. El-Beltagi, Usman Zulfiqar, Abdul Rehman, Gamal Awad El-Shaboury, Abdulrahman Alasmari, Orkhan Khudiyev, Mohd Asif Shah

**Affiliations:** a Department of Botany, University of Agriculture, Faisalabad, Pakistan; b Department of Agronomy and Horticulture, University of Nebraska-Lincoln, West Central Research Extension and Education Center, North Platte, NE, USA; c Agricultural Biotechnology Department, College of Agriculture and Food Sciences, King Faisal University, Al-Ahsa, Saudi Arabia; d Department of Agronomy, Faculty of Agriculture and Environment, The Islamia University of Bahawalpur, Bahawalpur, Pakistan; e State Key Laboratory of Efficient Utilization of Arid and Semi- Arid Arable Land in Northern China, Institute of Agricultural Resources and Regional Planning, Chinese Academy of Agricultural Sciences, Beijing, China; f Department of Biology, College of Science, King Khalid University, Abha, Saudi Arabia; g Department of Biology, Faculty of Science, University of Tabuk, Tabuk, Saudi Arabia; h Biodiversity Genomics Unit, Faculty of Science, University of Tabuk, Tabuk, Saudi Arabia; i Department of Basic Medical, Nakhchivan State University, Nakhchivan, Azerbaijan; j Kardan University, Kabul, Afghanistan; k Division of Research and Development, Lovely Professional University, Phagwara, Punjab, India; l University Centre for Research & Development, Chandigarh University, Gharuan, Mohali, Punjab, India

**Keywords:** Antioxidant, foliar spray, ionic imbalance, osmolyte protection, salt stress

## Abstract

Salinity stress poses a significant challenge to the productivity of maize, adversely affecting crop growth and development. The present study was conducted to elucidate the potential protective role of phenylalanine against salinity stress by modulating morphological traits, antioxidant defense mechanisms, and ionic balance. The study comprises two factors, including (i) salinity stress: NS = No salinity (control) SS = Salinity stress at 100 mM (sodium chloride), and phenylalanine foliar applications: NS = No spray, DWS = Distilled water spray, PAN10 = 10 mM, PAN20 = 20 mM, PAN30 = 30 mM, and PAN40 = 40 mM spray of phenylalanine. The results revealed that salinity stress caused a significant reduction in the morphological attributes (SFW by 47% and RFW by 39%) and photosynthetic pigments (chlorophyll a by 14% and chlorophyll b by 8%), while the foliar-applied PAN40 ameliorated the stress induce toxicity and improved these morphological attributes by 49% and 43% and photosynthetic pigments by 32% and 47%, respectively as compared to control conditions. Salinity stress increased the production of stress indicators such as MDA by 10% and H_2_O_2_ by 19%, while foliar application of PAN40 improved the activities of enzymatic and non-enzymatic antioxidants such as POD by 38%, CAT by 36%, and AsA by 38% and reduced the production of MDA by 10% and H_2_O_2_ by 19% under salinity stress. In crux, the foliar-applied PNA (40 mM) showed better performance under stress by improving the morphological and biochemical attributes to cope with the toxic effects of stress. The application of PAN may serve as a scalable, sustainable strategy to impart salinity stress tolerance in maize, improving both agronomic and biochemical resilience.

## Introduction

Maize (*Zea mays* L.) ranks as a leading global food, feed, and forage crop cultivated on nearly 190–200 million hectares worldwide, with annual production surpassing 1.2 billion metric tons.^1^ With the growing global population, shifts toward protein-rich diets and rising demand for animal products, maize production must double by roughly 2050 to ensure food security amid climate change and evolving land-use patterns.^2^ However, achieving this will expose crops to intensified abiotic stresses through both direct and indirect adverse effects.^3^ ^,^ ^4^ However, abiotic stresses such as drought and salinity, intensified by global climate change and water scarcity, now seriously threaten future maize production and supply.^5^

In recent decades, the expansion of saline–alkaline soils have accelerated as a result of climate change, posing a serious challenge to sustainable crop productivity.^6^ ^,^ ^7^ Maize is regarded as a crop with moderate sensitivity to salinity, and salt stress remains one of the most prevalent abiotic constraints limiting its productivity. Globally, soil salinization affects over 1000 million hectares, accounting for nearly 6%–7% of the Earth’s total land surface.^8^ Saline soils are primarily defined by excessive salt accumulation, particularly that of sodium chloride (NaCl). In glycophytic plant species, exposure to relatively low salinity levels can trigger stress responses; For instance, NaCl concentrations around 40 mmol L^−1^, corresponding to an osmotic potential of approximately −0.2 MPa, are sufficient to induce physiological stress.^9^ At higher concentrations, such as 100 mmol L^−1^ NaCl, maize seedling growth can be reduced by nearly half.^10^ Since nearly 70% of global maize cultivation occurs in arid and semi-arid regions, where salinity problems are widespread, soil salinization represents a major threat to maize production worldwide.^9^ Salt stress impairs maize performance through multiple interconnected mechanisms, including osmotic imbalance, excessive accumulation of toxic ions such as Na⁺ and Cl^−^, and enhanced oxidative damage.^11^ ^,^ ^12^ In general, salinity adversely affects seed germination, vegetative growth, and developmental processes by lowering osmotic potential, disturbing ionic equilibrium, restricting water uptake, impairing nutrient acquisition, and disrupting metabolic and microbial activities, ultimately leading to significant yield losses in many crops.^13–15^

To withstand saline environments, plants have developed diverse adaptive mechanisms involving osmotic adjustment, maintenance of ion homeostasis, detoxification of reactive oxygen species (ROS), and regulation of phytohormone signaling pathways.^16^ ^,^ ^17^ These adaptive responses enable plants to mitigate salt-induced damage through multiple defense strategies.^18^ Among these strategies, the activation of enzymatic and non-enzymatic antioxidant systems plays a crucial role in scavenging excess ROS and protecting cellular structures under stress conditions.^19–22^ In addition, plants synthesize a range of stress-related proteins and compatible solutes, including specific amino acids, which further contribute to salinity tolerance and stress resilience.

Phenylalanine (PAN) is a fundamental amino acid that plays a central role in both primary and secondary plant metabolism.^23^ ^,^ ^24^ It serves as a key metabolic entry point for the biosynthesis of a vast array of plant-derived compounds. Approximately 20%–30% of photosynthetically fixed carbon is funneled into the phenylpropanoid pathway, leading to the formation of phenolic constituents such as lignin and related phenols. Beyond their structural functions, many metabolites originating from PAN participate in plant defense mechanisms, cellular signaling, and protection against ultraviolet radiation.^25^ ^,^ ^26^

As a metabolic precursor, PAN contributes to the synthesis of diverse phenolic substances, including flavonoids, lignin, condensed tannins, benzenoids, and phenylpropanoid-derived volatiles, all of which enhance plant tolerance to adverse environmental conditions.^24^ Exogenous application of PAN, either as a seed pretreatment or foliar spray, has been shown to stimulate antioxidant capacity and promote plant growth in several crops. Beneficial effects have been reported in mustard (*Brassica campestris* L.),^27^ tomato (*Solanum lycopersicum* L.)^24^ ^,^ ^25^ (*Glycine max* L.)^28^ and cucumber (*Cucumis sativus* L.).^29^

Although several studies have examined the involvement of phenylalanine (PAN) in plant responses to various abiotic stresses, its potential role in enhancing salinity tolerance in maize remains insufficiently explored. Therefore, the present study was designed to investigate whether exogenous PAN application could mitigate the adverse effects of salt stress on maize plants. It was hypothesized that external supplementation with PAN would enhance maize resilience to salinity by improving physiological and biochemical performance. Accordingly, the specific objectives of this study were: (i) to assess the influence of PAN on maize growth and development under saline conditions through the evaluation of physicochemical traits, including photosynthetic pigment content, antioxidant enzyme activities, non-enzymatic antioxidant levels, and osmolyte accumulation; and (ii) to determine the impact of PAN on ionic balance by analyzing the distribution and accumulation of mineral ions in the roots and shoots of maize exposed to salinity stress.

## Materials and methods

The experiment investigated the alleviation of salinity-induced damage in maize through foliar phenylalanine (PAN) application. It followed a completely randomized design (CRD) with three replications. Seeds of the maize hybrid Hyb-357, obtained from the Department of Botany, University of Agriculture, Faisalabad, were sown in plastic pots (30 × 27 × 25 cm) containing 8 kg of sandy loam soil (EC 1.35 dS m^−1^, pH 7.7, organic matter 0.98%, available P 7.8 mg kg^−1^, available K^+^ 160 mg kg^−1^, water saturation 42%) collected from the Old Botanical Garden backyard at the university. Salinity stress (100 mM NaCl) was applied before sowing to establish a uniform saline environment throughout the experiment. Foliar PAN was sprayed uniformly (approximately 10–15 ml) on both sides of the leaf surface until it was properly wet with the help of a hand spray bottle once after 30 DAS of sowing (BBCH stage 30: stem elongation.^30^ ^,^ ^31^ Treatments are detailed in Table 1. Twelve seeds were sown per pot and thinned to eight plants at 10 DAS. Nutrient supply was provided via Haugland's solution.^32^

**Table 1.** The description of factors and treatments.

Factors	Treatments
Salinity stress	NS = No salinity (control) SS = Salinity stress at 100 mM (Sodium chloride)
Phenylalanine (PAN)	NS = No spray DWS = Distilled water spray PAN10 = 10 mM spray of phenylalanine PAN20 = 20 mM spray of phenylalanine PAN30 = 30 mM spray of phenylalanine PAN40 = 40 mM spray of phenylalanine
Variety = Hyb-357	
The experiment was conducted under a completely randomized design and three replications	
Total pots = 36, Stressed pots = 18 pots, Foliar spray was done at 10 mL spray per pot	

### Morphological parameters

Root and shoot lengths were measured using a measuring scale. The root and shoot fresh weights were promptly determined by using a digital balance. The samples were oven-dried at 72 °C for 48 h, and the root and shoot dry weights were measured. The number of leaves per plant and the internodal distance were calculated manually from all the replications. The length and width of the leaves were measured on a meter scale, and then the sum was multiplied by 0.68 (correction factor) for cereals. The root and shoot diameters were measured using the Vernier Caliper.

### Biochemical parameters

#### Photosynthetic pigments

Photosynthetic pigments were extracted from 0.1 g fresh leaf tissue homogenized in 5 mL 80% (v/v) acetone. The samples were incubated overnight at 4 °C in the dark to maximize extraction efficiency, followed by measurement of absorbance at 663 nm (Chl a), 645 nm (Chl b), and 480 nm (carotenoids) with a spectrophotometer. Concentrations were determined according to the equations proposed by Arnon.^33^

#### Stress indicators

MDA was quantified by homogenizing 0.25 g leaves in 2 mL 0.1% TCA, centrifuging (1500 rpm, 20 min), mixing 1 mL supernatant with 4 mL 0.5% TBA in 20% TCA, heating at 95 °C (30 min), and measuring A₅₃₂ and A₆₀₀ after cooling. H₂O₂ content followed the method of Velikova,^34^ 0.25 g leaves extracted in 2 mL cold 0.1% TCA, centrifuged (1500 rpm, 20 min), and then 0.5 mL supernatant + 0.5 mL phosphate buffer + 1 mL 1 M KI, with the absorbance at 390 nm.

#### Antioxidant contents

The antioxidant enzyme activities were determined using standard protocols. Superoxide dismutase (SOD) activity was measured following Giannopolitis and Ries^35^ using a reaction mixture containing nitroblue tetrazolium, riboflavin, L-methionine, phosphate buffer, Triton X, distilled water, and an enzyme extract; the absorbance was recorded at 560 nm after 20 min of illumination. Peroxidase (POD) activity was assayed according to Chance and Maehly^36^ using guaiacol and hydrogen peroxide as substrates, with changes in absorbance monitored at 470 nm at 0, 30, 60, and 90 s. Catalase (CAT) activity was determined following the same method by measuring the decomposition of hydrogen peroxide at 240 nm at identical time intervals after mixing the enzyme extract with phosphate buffer and H₂O₂.

#### Non-enzymatic antioxidant activities

The total flavonoid content was determined following Marinova^37^using ethanolic extracts reacted with sodium nitrate, aluminum chloride, and 1 M NaOH, with the absorbance measured at 510 nm. The anthocyanin content was estimated according to Murray and Hackett^38^ by extracting 0.2 g of leaf tissue in acidified methanol (methanol:HCl, 120:1, v/v), incubating at 50 °C for 60 min, and recording the absorbance at 535 nm. Endogenous ascorbic acid was quantified following Mukherjee and Choudhuri^39^ after extraction in 6% trichloroacetic acid and reaction with acidic dinitrophenyl hydrazine and thiourea, followed by sulfuric acid addition; the absorbance was measured at 530 nm.

#### Osmolytes

Proline was quantified by homogenizing 0.25 g fresh leaves in 3% sulfosalicylic acid, filtering, and then reacting 1 mL extract with acid ninhydrin and glacial acetic acid at 100 °C (90 min); the absorbance was measured at 520 nm after cooling. Glycine betaine was measured by extracting 0.25 g fresh tissue in distilled water, centrifuging (12,000 rpm, 15 min), mixing 500 µL supernatant with 2 N H₂SO₄ and KI₃, incubating on ice (90 min), extracting into 1,2-dichloroethane, and reading the absorbance at 365 nm in the lower layer.

#### Ion analysis

Oven-dried root and shoot samples (0.1 g) were digested separately in 5 mL concentrated H₂SO₄ overnight, followed by heating with the gradual addition of H₂O₂ until a clear solution was obtained. After cooling, the digests were filtered and diluted to 50 mL with distilled water. Potassium and calcium concentrations were measured using a flame photometer. The nitrate content was determined following Kowalenko and Lowe^40^ by reacting 3 mL of the digest with 7 mL of chromotropic acid reagent, and the absorbance was recorded at 430 nm.

### Statistical analysis

A completely randomized design was employed to create three replications of the experiment (CRD). The recorded data were analyzed at a 5% significance level using Statistics software version 8.1. Furthermore, the figures were made with Microsoft Excel (2016 edition) (Microsoft Corporation, Redmond, Washington, USA).

## Results

### Morphological attributes

Salinity stress and foliar application of phenylalanine significantly affect the morphological attributes of maize plants. It was observed that salinity stress (100 mM) caused a significant decline in morphological attributes, such as shoot fresh and dry weight by 47% and 48%, root fresh and dry weight by 39% and 35%, shoot and root length by 9% and 48%, and stem diameter by 19%, as compared to the control (Table 2). On the other hand, foliar application of phenylalanine produced stimulatory effects on morphological indices under saline and non-saline (control) conditions. Specifically, phenylalanine ameliorated the toxicity of salinity stress and improved the above-mentioned morphological indices as compared to the control. Among the other applications of phenylalanine, foliar application at 40 mM showed the highest increase in morphological attributes, such as shoot fresh weight by 49%, and root fresh weight by 43%, respectively, under salinity stress as compared to the control (no-foliar application).

**Table 2.** Impact of foliar applied phenylalanine (PAN) on the morphological attributes of maize plant under control and salinity stress conditions.

Salinity stress	Treatments	SWF (g)	RWF (g)	SDW (g)	RDW (g)	SL (cm)	RL (cm)	SD (cm)
NS (no-stress)	NS	5.5 ± 0.2g	2.1 ± 0.2e	1.67 ± 0.2g	0.85 ± 0.2g	10 ± 0.2g	2.55 ± 0.2h	3.4 ± 0.2e
	DWS	7 ± 0.04ef	2.9 ± 0.04c	2.5 ± 0.04e	1.2 ± 0.04f	15 ± 0.04e	3.7 ± 0.04fg	3.7 ± 0.04e
	PAN10	8.3 ± 0.2cd	3.2 ± 0.2c	3.5 ± 0.2c	1.4 ± 0.2e	18 ± 0.2cd	4.7 ± 0.2d	5.66 ± 0.2c
	PAN20	9.9 ± 0.3b	3.6 ± 0.3b	3.789 ± 0.3bc	1.8 ± 0.3c	20 ± 0.3c	6.2 ± 0.3c	7.66 ± 0.3b
	PAN30	10.3 ± 0.3b	3.9 ± 0.3b	4.1 ± 0.3b	2.1 ± 0.3b	26.3 ± 0.3a	7.3 ± 0.3b	8.99 ± 0.3a
	PAN40	12.3 ± 0.4a	4.5 ± 0.4a	5.1 ± 0.4a	2.7 ± 0.4a	28.3 ± 0.4a	8.4 ± 0.4a	9.65 ± 0.4a
SS (salinity stress)	NS	4.1 ± 0.05h	1.8 ± 0.05e	1.45 ± 0.06g	0.65 ± 0.06h	8.1 ± 0.06g	2.2 ± 0.06h	2.2 ± 0.05f
	DWS	6.1 ± 0.05fg	2.0 ± 0.05e	1.78 ± 0.05fg	0.76 ± 0.05gh	12.6 ± 0.05f	3.4 ± 0.05g	3.4 ± 0.05e
	PAN10	6.8 ± 0.3ef	2.1 ± 0.3e	2.1 ± 0.3f	1.2 ± 0.3f	16 ± 0.3de	3.9 ± 0.3efg	4.5 ± 0.3d
	PAN20	7.6 ± 0.5de	2.5 ± 0.5d	2.7 ± 0.5e	1.4 ± 0.5e	18 ± 0.5cd	4 ± 0.5ef	5.5 ± 0.5c
	PAN30	8.8 ± 0.3c	2.9 ± 0.3c	2.788 ± 0.3de	1.6 ± 0.3d	22.3 ± 0.3b	4.2 ± 0.3def	7.7 ± 0.3b
	PAN40	10 ± 0.1b	3.2 ± 0.1c	3.1 ± 0.1 d	1.8 ± 0.1c	27 ± 0.1a	4.4 ± 0.1de	8.1 ± 0.1b

NS (No-spray), DWS (distilled water spray), PAN10 (10 mM spray of phenylalanine), PAN20 (20 mM spray of phenylalanine), PAN30 (30 mM spray of phenylalanine), and PAN40 (40 mM spray of phenylalanine). Shoot fresh weight (SFW), root fresh weight (RFW), shoot dry weight (SDW),root dry weight (RDW), shoot length (SL), and root length (RL). Different letters after the values (mean of three replicates ± standard error) indicate significant differences at *p* < 0.05 according to the Tukey HSD test.

#### Photosynthetic pigments

**Figure 1.**
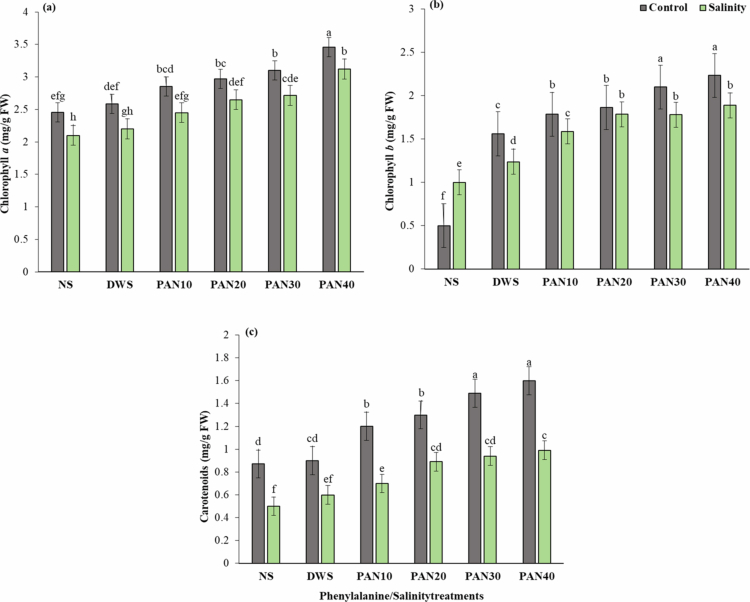
Effect of foliar-applied phenylalanine (PAN) on the (a) chlorophyll a, (b) chlorophyll b, and (c) carotenoid contents of the maize plant under control and salinity stress conditions. The error bars above the mean (three replicates) showed standard error, and the different letters indicate significant differences (at *p* < 0.05) according to the Tukey HSD test.

Salinity stress (100 mM NaCl) substantially decreased photosynthetic pigments in maize, with reductions of 14% in chlorophyll a, 8% in chlorophyll b, and 59% in carotenoids compared to the control. Conversely, foliar phenylalanine application improved pigment contents in both control and salt-stressed plants (Figure 1). The 40 mM phenylalanine treatment was most effective under salinity, enhancing chlorophyll a by 32%, chlorophyll b by 47%, and carotenoids by 49% relative to the untreated saline control.

## Stress indicators production

**Figure 2.**
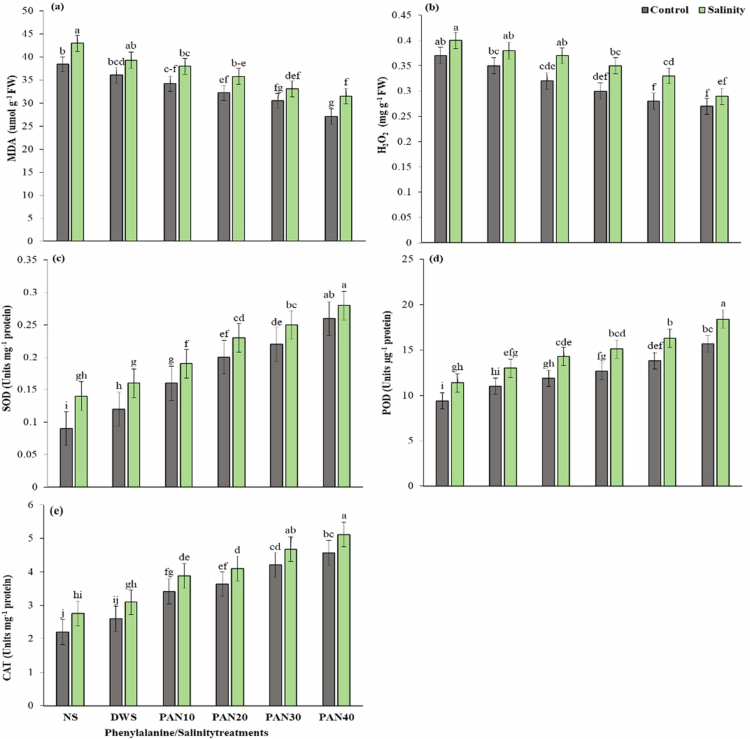
Effect of foliar-applied phenylalanine (PAN) on the (a) MDA, (b) H_2_O_2_, (c) SOD, (d) POD, and (e) CAT contents of the maize plant under control and salinity stress conditions. The error bars above the mean (three replicates) showed standard error, and different letters indicate significant differences (at *p* < 0.05) according to the Tukey HSD test.

The foliar application of phenylalanine and salinity stress showed an impact on the stress indicators of maize plants. It was found that salinity stress (100 mM) caused an increase in the production of stress indicators, such as H_2_O_2_ and MDA, by 11% and 10%, respectively as compared to control (no-saline) conditions. The foliar application of phenylalanine takes part in reducing the overproduction of these stress indicators to cope with oxidative damage. Among the other foliar applications of phenylalanine, the foliar-applied (40 mM) showed a significant reduction in the level of H_2_O_2_ by 37% and MDA by 36% under salinity stress as compared to the no-foliar application (Figure 2).

### Enzymatic antioxidants activities

The salinity stress and foliar application of phenylalanine significantly affect the activity of enzymatic antioxidants. Salinity stress (100 mM) significantly increased the antioxidant activities such as SOD, POD, and CAT, by 20%, 15%, and 12%, respectively, as compared to the control conditions. This increase in the above-mentioned antioxidant activity was not enough to cope with the oxidative damage (Figure 2). On the other hand, foliar application of phenylalanine plays a vital role in improving the antioxidant activity under both saline and non-saline circumstances. Among the other applications of phenylalanine, the foliar application (40 mM) showed a greater increase in the activity of SOD by 64%, POD by 38%, and CAT by 46% under salinity stress as compared to no-foliar application.

### Non-enzymatic antioxidants activities

**Figure 3.**
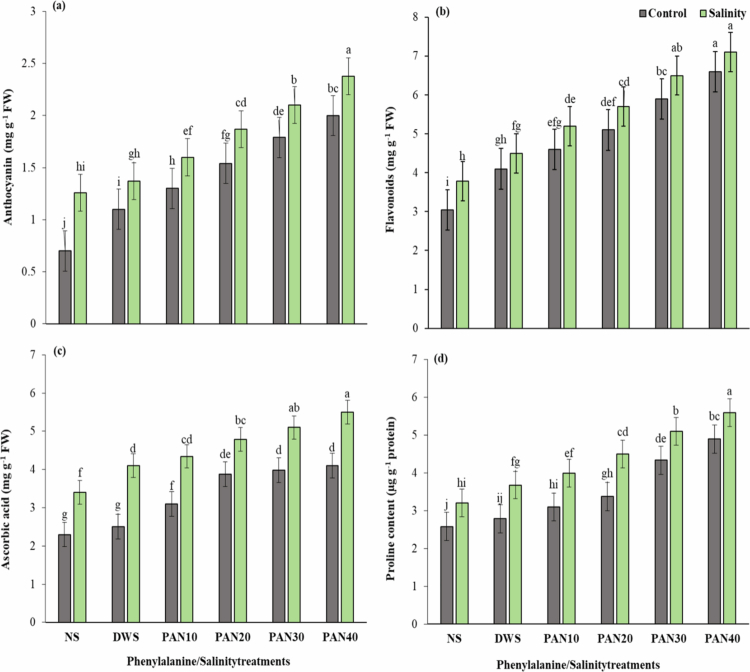
Effect of foliar-applied phenylalanine (PAN) on the (a) anthocyanin, (b) flavonoid, (c) ascorbic acid, and (d) proline contents of the maize plant under control and salinity stress conditions. The error bars above the mean (three replicates) showed standard error, and different letters indicate significant differences (at *p* < 0.05) according to the Tukey HSD test.

Salinity increased the activity of non-enzymatic antioxidants in maize. In the current study, salt stress at 100 mM enhanced non-enzymatic antioxidants such as flavonoids by 10%, anthocyanin by 20%, and ascorbic acid by 27% when compared to the control conditions. The foliar application of phenylalanine significantly improved non-enzymatic antioxidant activity under salinity stress and control conditions. Among the other foliar applications of phenylalanine, foliar spray at 40 mM significantly increased the activity of non-enzymatic antioxidant flavonoid by 46%, anthocyanin by 47%, and ascorbic acid by 38% under salt stress as compared to the control with no foliar application (Figure 3).

### Osmoprotectant production

Salinity stress and foliar application of phenylalanine showed a significant effect on the osmoprotectants in maize plants. Salinity (100 mM) significantly increased the osmoprotectant content of maize, such as proline, by 19% compared to the control. On the other hand, foliar-applied phenylalanine significantly improved the osmoprotectant levels under both salinity stress and control conditions. Among the foliar applications of phenylalanine, foliar spray at 40 mM positively increased the osmoprotectant content as proline, by 42% during salinity stress when compared to the control (Figure 3).

### Ionic homeostasis

**Figure 4.**
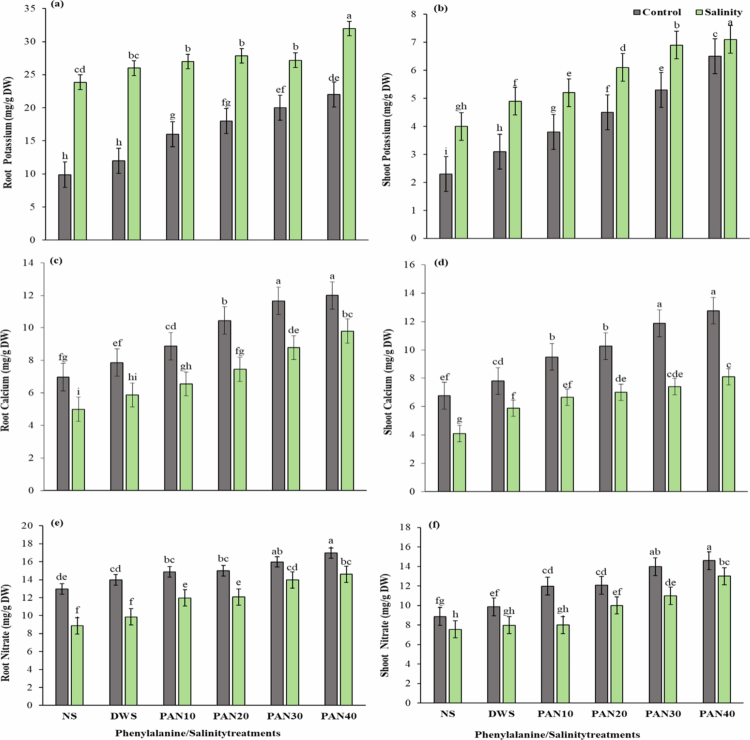
Effect of foliar-applied phenylalanine (PAN) on the (a) root potassium, (b) shoot potassium, (c) root calcium, (d) shoot calcium, (e) root nitrate, and (f) shoot nitrate of maize plants under control and salinity stress conditions. The error bars above the mean (three replicates) showed standard error, and different letters indicate significant differences (at *p* < 0.05) according to the Tukey HSD test.

Salinity significantly reduced the ion content of maize. It was discovered that in the current study, salinity stress of 100 mM reduced the ion content, such as root and shoot potassium, by 40% and 25%, root and shoot calcium, by 32% and 30%, and root and shoot nitrate, by 25% and 24% when compared to the control (Figure 4). On the other hand, foliar application of phenylalanine had stimulatory effects on the ion content under both saline and non-saline situations. Among the phenylalanine applications, foliar spray at 40 mM demonstrated the most significant increase in the ion content, as the root and shoot potassium by 25% and 43%, the root and shoot calcium by 50% and 59%, and the root and shoot nitrate by 39% and 41%, respectively, under salinity stress as compared to the control (no-foliar spray).

### Heat map and principal component analysis

**Figure 5.**
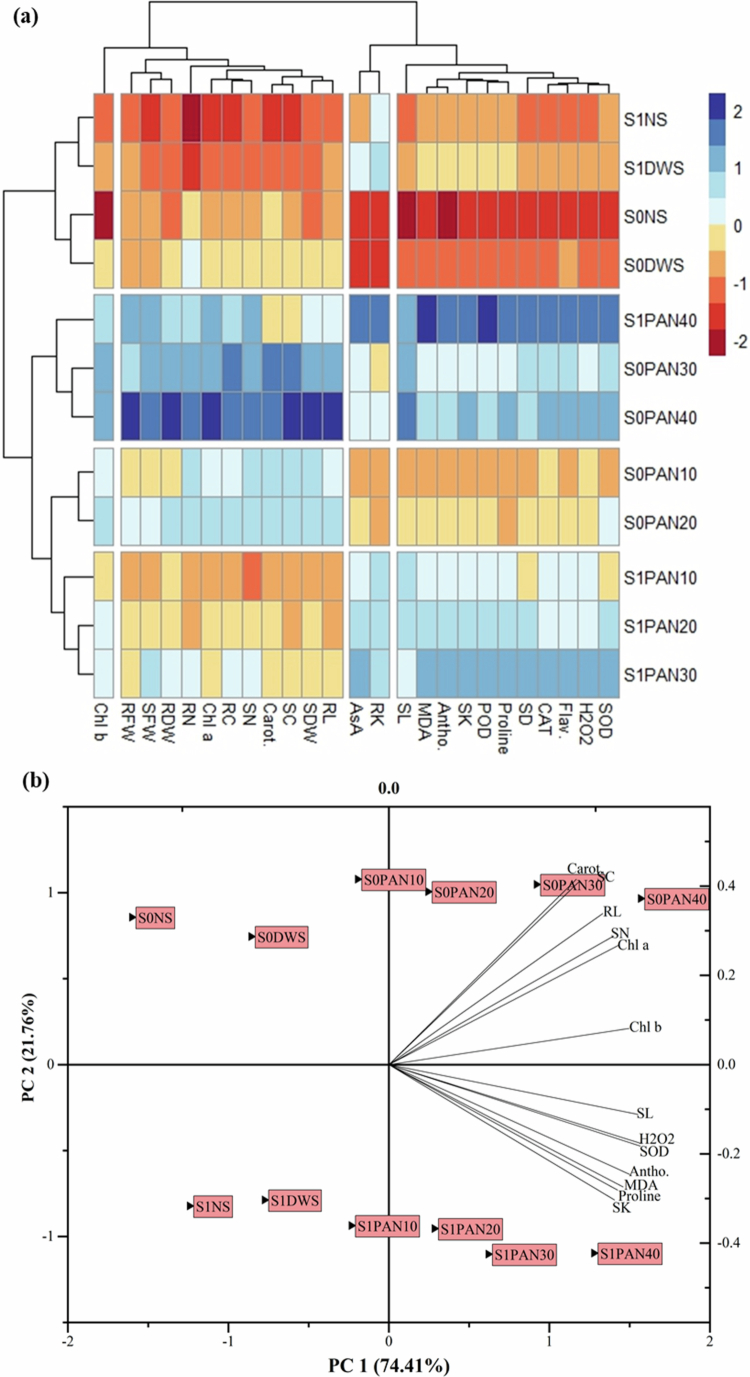
Heat map analysis (a) and principal component analysis (b) across the recorded attributes of maize under the influence of foliar-applied phenylalanine and salinity stress. The abbreviations of the attributes are SFW (shoot fresh weight), SDW (shoot dry weight), RFW (root fresh weight), RDW (root dry weight), SL (shoot length), RL (root length), SD (stem diameter), Chl. a (chlorophyll a), Chl. b (Chlorophyll b), Carot. (carotenoids), MDA (malondialdehyde), H_2_O_2_ (hydrogen peroxide), SOD (superoxide dismutase), POD (peroxidase), Flav. (flavonoids), Antho. (anthocyanin), AsA. (Ascorbic acid), Proline, RK (root potassium), SK (shoot potassium), RC (root calcium), SC (shoot calcium), NR (root nitrate), and SN (shoot nitrate).

Heat map analysis (Figure 5) evaluated morphological, photosynthetic, biochemical, and ionic traits under salinity and phenylalanine treatments. The colors ranged from blue (strongly positive/increased) to red (strongly negative/decreased), with the intensity showing interaction strength. The highest positive responses occurred in growth indices, pigments, antioxidants, and ionic contents under S₀PAN40 and S₁PAN40. Significant declines were noted under S₁NS (salinity without foliar application).

## Discussion

In arid and semiarid areas of the world, salinity is the major threat to agriculture, which disturbs the plant's physiological as well as biological activities, imbalances nutrient uptake, and reduces plant biomass, which ultimately causes food shortages around the world.^41^ However, this experiment supports that foliar application of PAN modifies plant attributes by enhancing the antioxidative system, enzymatic activities, and photosynthetic activities, and increasing plant biomass, which helps combat salinity stress. The experimental results support our hypothesis that foliar applications of PAL reduce the negative effects of salinity stress in maize by upregulating the plant defense system. Results have revealed that salinity stress reduced the plant growth and development of maize by disturbing the plant's physical attributes, including their photosynthetic pigments, photosynthetic rate, gas exchange, and water status of the maize plants. However, foliar application of PAN alleviated the toxic effects of salinity stress by improving plant growth and development due to the upregulation of antioxidant activities, osmolyte production, and the reduction in the production and activities of stress indicators, such as H_2_O_2_, MDA, and electrolyte leakage (Table 2 and Figures 1–4).

The results of this study showed a significant reduction in plant growth attributes, including above-ground and below-ground parameters, such as plant height, leaf fresh and dry weight, root length, and root fresh and dry weight, in maize plants. The results of the experiment showed that root and shoot length are negatively impacted by salinity stress, which is correlated with the effects on cucumber plants.^29^ ^,^ ^42^ Furthermore, it also stunted the growth rate, which decreases the leaf size, weight, as well as internodal length, and stem diameter.^43^ Consequently, higher levels of salts in the soil negatively impact the growth, cell division, metabolism, and yield of plants.^44^ However, PAN supplementation played an important role in reducing the deleterious effects of salinity stress in maize plants. The plant growth parameters were improved by PAN applications under stressed conditions, and maximum improvement was observed at PAN20.

Moreover, the rising level of salts in the soil reduces the soil water potential, which inhibits the root's water absorption, leading to a water deficit in plants.^45–47^ Deficiency of water negatively affects stomatal conductance, which reduces the rate of carbon fixation and assimilation, resulting in plant biomass as well as yield reduction.^48^ Salinity stress also reduces photosynthesis in plants, which is one of the growth-determining factors because of water imbalance, which disturbs stomatal conductance. Stomatal closure decreases the intake of CO_2,_ which results in a lower concentration of assimilated production.^49^ ^,^ ^50^ Phenylalanine is an organic compound containing nitrogen, which is a basic component of enzymes as well as proteins, so the exogenous application of this compound increases crop growth and crop yield by improving photosynthates, reducing the effects of water deficit by balancing the osmotic potential, enhancing the absorption of nutrients, minerals uptakes, and IAA, as recorded in Moringa oleifera and maize.^27^ ^,^ ^51^ Moreover, in this experiment observed that PAN application increased the total nitrogen level in plants, similar to other amino acid applications, such as glycine, glutamate, and cysteine.^28^ Its foliar spray also raised the carotenoids,^25^ which reduces the photo-oxidative damage risks as well as increases the growth rate.^52^

Saline stress induces the production of chlorophyllase enzymes, which degrade the chlorophyll content, as well as disorganize the thylakoid membranes. The reduction in chlorophyll disturbs the photosynthetic rates, which consequently deteriorates plant health and decreases productivity.^25^ ^,^ ^53^ In this trial, higher levels of salts in the soil inhibited root development, accompanied by reduced water and nutrient absorption.^54^ Excessive Na^+^ concentration in the root zone develops competition at binding sites for Na^+^ and K^+^, which inhibits K^+^ absorption.^55^ Further, it also depresses the K^+^ and P levels in the leaves, which are correlated with moringa leaves.^56^

Plants tend to compartmentalize the Cl^−^ or Na^+^ ions in vacuoles to tolerate stress, but at very high salt concentrations of these ions, after the saturation of vacuoles start to move into the cytoplasm which resulting in the inhibition of enzymes, cellular dehydration, as well as plunged seed germination.^57^ Additionally, the results concluded that the rising levels of salts reduce the free amino acid content; however, PAN application increases these amino acid concentrations. Moreover, it also regulates and synthesizes many enzymes and genes, which are the signals for the synthesis of various amino acids, and is also involved in the maintenance of redox homeostasis, which is helpful for salt tolerance in plants.^58^ Further, it also decreases the activity of POD and CAT, which decreases the production of ROS to combat stressful environments.^25^

In a stressful environment, plants change their metabolism, which decreases plant growth and yield with the increase in the concentration of secondary metabolites such as osmolytes, terpenoids, fatty acids, and antioxidant compounds.^56^ Salinity stress is recorded to increase the activity of non-enzymatic (flavonoids, vitamin C, and phenolic compounds) as well as enzymatic antioxidant activities.^59–61^ Phenylalanine treatment has a crucial importance in forming these secondary metabolites, which are reported to protect the cells from oxidative stress by increasing membrane stabilization.^62^ Furthermore, by PAN application, the increasing level of these secondary metabolites also regulates the metabolic reactions of the plants, which are correlated with the results that were recorded in a previous rise in phenolic concentrations in plants^63^ ^,^ ^64^ as well as flavonoids.^65^ The phenolic compounds act as hydrogen donors and deactivators for singlet oxygen, which helps scavenge the ROS.^66^ PAN may improve tolerance to salinity by controlling several signaling pathways that respond to stress. PAN, the main precursor of the phenylpropanoid pathway, stimulates the production of lignin, flavonoids, and phenolic compounds that increase antioxidant capacity and guard cellular structures from oxidative damage.^67^

Additionally, the application of PAN interconnects primary as well as secondary metabolism, playing the role of a precursor for several compounds that are essential for the growth and reproduction of plants in stressful environments.^68^ It also increases the proline content, which is responsible for inducing the stress-responding genes, quenching of ROS, lower leakage of electrolytes, and membrane stability to tolerate salt stress.^25^ Phenylalanine also induces PAN ammonia-lyase as well as chalcone synthase, which regulates the phenolic synthesis in stressful environments. Teixeira^69^ and Sadak^70^ also recorded that its foliar application, like other amino acids, increased the antioxidant activities. Phenylalanine is also a derivative of phenylpropanoid, as well as associated with numerous compounds like suberin, cutin, lignin, lignans, and tannins, which are the constituents of the cell wall protecting against ultraviolet light and abiotic stress.^68^ Further foliar spray of PAN contributes to stress tolerance by stabilizing the plasma membrane from stress-induced damage and protecting photosystems as well as pathways related to secondary metabolism.^25^

Furthermore, ionic toxicity is also responsible for damaging the membranous system which is treated with the application of amino acids like PAN by increasing the Ca^2+^ concentration within the cytoplasm by depolarizing the membrane.^71^ It was observed that the application of PAN increases the assimilation of nitrogen and carbon, stabilizes the membranous components in stressful environments, which increases growth.^72^ Increasing concentrations of MDA in saline stress act as an indicator of membrane damage from ROS by inducing lipid peroxidation, which is positively related to the extent of membrane damage.^25^ The results of PAN foliar spray are similar to other reports that it decreases the contents of MDA, which is an indicator of membrane protection from oxidative damage.^73^

The imbalance of nutrient uptake for increasing concentrations of Cl^−^ and Na^+^ due to salinity also reduces the plant biomass. Previous reports showed that these increasing ionic concentrations stacked up in the plants’ vegetative parts, leading to ionic toxicity.^29^ ^,^ ^74^ This salt stress was also recorded as a factor that limited the growth and biomass yield.^75^ ^,^ ^76^ Exogenous application of PAN in such a situation improves the uptake of ions like Ca^2+^, K^+^, and Mg^2+,^ which are essential for increasing nutrient uptake and plant biomass.^29^ Additionally, amino acid applications like PAN spray decrease the level of Na^+^ by increasing the K^+^ and P content in plant cells or simply boosting the macronutrient concentrations in plants.^77^ By influencing stress-responsive signaling pathways involved in the regulation of Na^+^ and K^+^ transporters, PAN may help preserve ionic homeostasis by lowering Na^+^ toxicity and enhancing K^+^ retention in saline environments.^78^ In salt stress, PAN application successfully increases the carbohydrate level, which is responsible for improving plant growth.^25^ ^,^ ^79^ Further, its usage also increases total soluble salts in plants, performing osmoprotective functions by increasing water and nutrient uptake, which modifies plants' metabolism and photosynthesis.^56^ ^,^ ^66^

Although the current results show that PAN improves salt tolerance, it's crucial to keep in consideration that its effects might vary depending on concentration. Increased PAN concentrations could worsen ionic imbalance in saline environments by increasing metabolic demands, disrupting amino acid homeostasis, or altering ion uptake and transport. If the optimal threshold is surpassed, these impacts may decrease plant growth and nutrient uptake.^80^ The beneficial reactions reported at the tested concentrations in the current study indicate that PAN acted within a desirable physiological range; however, more research is needed to determine the upper safe limit of PAN application under various environmental conditions. Optimizing PAN's agricultural application under salt stress will require an understanding of its concentration-dependent effects.

## Conclusion

According to this study, by strengthening physiological and biochemical defense systems, exogenous phenylalanine (PAN) can reduce salinity-induced damage and increase salt tolerance in maize. Among all the other foliar treatments, PAN (40 mM) performed better in improving the growth of the plants in salinity by protecting their photosynthetic pigments, enhancing enzymatic and non-enzymatic antioxidant mechanisms to reduce ROS-induced oxidative damage, promoting osmolyte production, and maintaining ionic homeostasis. These results show that PAN is a viable, sustainable, and useful method for enhancing maize performance under salt-affected conditions. It may also be used in the field in the future as an affordable foliar treatment. However, more research is needed to understand the molecular mechanisms of PAN-mediated salt tolerance. To better understand the underlying regulatory networks and support the suggested mechanism of PAN-induced salinity tolerance, future research should confirm the expression of key salt-responsive genes that contribute to phenylpropanoid metabolism (e.g., PAL), ROS signaling, antioxidant regulation, and ion transport.

## Data Availability

Data will be made available upon request.

## References

[1] Ali B , Hafeez A , Afridi MS , Javed MA , Sumaira , Suleman F , Nadeem M , Ali S , Alwahibi MS , Elshikh MS , et al. Bacterial-mediated salinity stress tolerance in maize (*Zea mays* L.): a fortunate way toward sustainable agriculture. ACS Omega. 2023;8(23):20471–20487. doi: 10.1021/acsomega.3c00723. 37332827 PMC10275368

[2] Karapetsas N , Gobin A , Bilas G , Koutsos TM , Pavlidis V , Katragkou E , Alexandridis TK . Analysis of land suitability for maize production under climate change and its mitigation potential through crop residue management. Land. 2024;13(1):63. doi: 10.3390/land13010063.

[3] Naveed M , Ditta A , Ahmad M , Mustafa A , Ahmad Z , Conde-Cid M , Tahir S , Shah SAA , Abrar MM , Fahad S . Processed animal manure improves morpho-physiological and biochemical characteristics of *Brassica napus* L. Under nickel and salinity stress. Environ Sci Pollut Res. 2021;28(33):45629–45645. doi: 10.1007/s11356-021-14004-3. 33871777

[4] Rahim HU , Ali W , Uddin M , Ahmad S , Khan K , Bibi H , Alatalo JM . Abiotic stresses in soils, their effects on plants, and mitigation strategies: a literature review. Chem Ecol. 2025;41(4):552–585. doi: 10.1080/02757540.2025.2481648.

[5] Ali B , Rehman A , Latef AAHA , Imran M , Javed MA , Wise N , Imin N . Engineering abiotic stress tolerance in cereal crops: current advances and future directions. Cereal Res Commun. 2026:804. doi: 10.1007/s42976-025-00578-3.

[6] Wu H . Plant salt tolerance and Na^+^ sensing and transport. Crop J. 2018;6(3):215–225. doi: 10.1016/j.cj.2018.01.003.

[7] Fareed S , Haider A , Ramzan T , Ahmad M , Younis A , Zulfiqar U , Rehman HU , Waraich EA , Abbas A , Chaudhary T , et al. Investigating the growth promotion potential of biochar on pea (*Pisum sativum*) plants under saline conditions. Sci Rep. 2024;14(1):10870. doi: 10.1038/s41598-024-61394-6. 38740776 PMC11091058

[8] Ivushkin K , Bartholomeus H , Bregt AK , Pulatov A , Kempen B , de Sousa L . Global mapping of soil salinity change. Remote Sens Environ. 2019;231:111260. doi: 10.1016/j.rse.2019.111260.

[9] Cao Y , Zhou X , Song H , Zhang M , Jiang C . Advances in deciphering salt tolerance mechanism in maize. Crop J. 2023;11(4):1001–1010. doi: 10.1016/j.cj.2023.03.009.

[10] Zhang Y , Li Y , Liu H , Xie H , Liu J , Hua J , Xiong M , Song H , Yong C . Effect of exogenous melatonin on corn seed germination and seedling salt damage mitigation under NaCl stress. Plants. 2025;14(7):1139. doi: 10.3390/plants14071139. 40219206 PMC11991619

[11] Islam M , Islam M , Hasan M , Hafeez ASMG , Chowdhury M , Pramanik M , Iqbal M , Erman M , Barutcular C , Konuşkan Ö , et al. Salinity stress in maize: consequences, tolerance mechanisms, and management strategies. OBM Genet. 2024;8(2). doi: 10.21926/obm.genet.2402265.

[12] He X , Zhu J , Gong X , Zhang D , Li Y , Zhang X , Zhao X , Zhou C . Advances in deciphering the mechanisms of salt tolerance in maize. Plant Signal Behav. 2025;20(1):2479513. doi: 10.1080/15592324.2025.2479513. 40098499 PMC11959903

[13] Askari-Khorasgani O , Rehmani MIA , Wani SH , Kumar A . Osmotic stress: an outcome of drought and salinity. Handbook of Plant and Crop Physiology. Boca Raton (FL): CRC Press; 2021; p. 445–464.

[14] Ahmed M , Tóth Z , Decsi K , et al. The Critical Role of Jasmonic Acid to Induce Salt Tolerance and Improve Crop Productivity: Review and Prospective. J Soil Sci Plant Nutr. 2025;25(5):3584–3602. doi: 10.1007/s42729-025-02354-7.

[15] Qasim F , Jamil M , Ahmed M , et al. Co-application of biochar, vermicompost and silicon mitigates salinity stress and enhances maize productivity in saline-calcareous soils Biodegradation. 2026;37:88. 10.1007/s10532-026-10308-8. 42142148

[16] Ahmad M , Waraich EA , Zulfiqar U , Ullah A , Farooq M . Thiourea application increases seed and oil yields in camelina under heat stress by modulating plant water relations and antioxidant defense system. J Soil Sci Plant Nutr. 2023;23(1):290–307. doi: 10.1007/s42729-022-01030-5.

[17] Ma L , Li J , Li J , Huo Y , Yang Y , Jiang C , Guo Y . Plant salt-tolerance mechanisms: classic signaling pathways, emerging frontiers, and future perspectives. Mol Plant. 2026;19(3):538–570. doi: 10.1016/j.molp.2025.12.003. 41403128

[18] Ilyas M , Maqsood MF , Shahbaz M , Zulfiqar U , Ahmad K , Naz N , Ali MF , Ahmad M , Ali Q , Yong JWH , et al. Alleviating salinity stress in canola (*Brassica napus* L.) through exogenous application of salicylic acid. BMC Plant Biol. 2024;24(1):611. doi: 10.1186/s12870-024-05473-3. 38926637 PMC11210054

[19] Ahmad M , Waraich EA , Hussain S , Zulfiqar U , Teshome FT , Gastelbondo M , Imran M , Farooq M . Exogenous application of thiourea improves the growth, seed yield, and seed fatty acid profile in late sown camelina. J Soil Sci Plant Nutr. 2023;23(1):1306–1325. doi: 10.1007/s42729-022-01074-7.

[20] Ahmad M , Waraich EA , Shahid H , Ahmad Z , Zulfiqar U , Mahmood N , Al-Ashkar I , Ditta A , El Sabagh A . Exogenously applied potassium enhanced morpho-physiological growth and drought tolerance of wheat by alleviating osmotic imbalance and oxidative damage. Pol J Environ Stud. 2023;32(5):4581–4595. doi: 10.15244/pjoes/163694.

[21] Ahmad M , Waraich EA , Zulfiqar U , Yong JWH , Ishfaq M , Din KU , Ullah A , Abbas A , Awan MI , Moussa IM , et al. Thiourea improves yield and quality traits of *Brassica napus* L. By upregulating the antioxidant defense system under high-temperature stress. Sci Rep. 2024;14(1):12195. doi: 10.1038/s41598-024-63064-z. 38806561 PMC11133410

[22] Ahmad M , Waraich EA , Munir A , Hussain S , Ahmed R , Iqbal MA , Zulfiqar U , Almutairi KF . Mitigating drought by exogenous potassium-mediated improvements in water relation, antioxidant defense, morpho-physiological, and biochemical attributes of black gram (*Vigna mungo* [L.] Hepper). Legume Res. 2024. doi: 10.18805/LRF-830.

[23] Bakhoum GS , Badr EAE , Sadak MS , Kabesh MO , Amin GA . Improving growth, some biochemical aspects and yield of three cultivars of soybean plant by methionine treatment under sandy soil condition. Int J Environ Res. 2019;13:35–43. doi: 10.1007/s41742-018-0159-2.

[24] Kumar V , Nadarajan S , Boddupally D , Wang R , Bar E , Davidovich-ikanati R R , Doron-Faigenboim A , Alkan N , Lewinsohn E , Elad Y , Oren-Shamir M , et al. Phenylalanine treatment induces tomato resistance to Tuta absoluta via increased accumulation of benzenoid/phenylpropanoid volatiles serving as defense signals. Plant J. 2024;119(1):84–99. doi: 10.1111/tpj.16745. 38578218

[25] Almas HI , Un- Anwar NZ , Kausar S , Farhat A , Munawar F , Khalizadieh M . R. Exogenous application of methionine and phenylalanine confers salinity tolerance in tomato by concerted regulation of metabolites and antioxidants. J Soil Sci Plant Nutr. 2021;21(4):3051–3064. doi: 10.1007/s42729-021-00598-0.

[26] Wu X , Zhu S , He L , Cheng G , Li T , Meng W , Wen F . Phenylalanine ammonia-lyase: a core regulator of plant carbon metabolic flux redistribution-from molecular mechanisms and growth modulation to stress adaptability. Plants. 2025;14(24):3811. doi: 10.3390/plants14243811. 41470693 PMC12737037

[27] Ramzan T , Shahbaz M , Maqsood MF , Zulfiqar U , Saman RU , Lili N , Irshad M , Maqsood S , Haider A , Shahzad B , et al. Phenylalanine supply alleviates drought stress in mustard (*Brassica campestris*) by modulating plant growth, photosynthesis, and antioxidant defense system. Plant Physiol Biochem. 2023;201:107828. doi: 10.1016/j.plaphy.2023.107828. 37329687

[28] Teixeira WF , Fagan EB , Soares LH , Soares JN , Reichardt K , Neto DD . Seed and foliar application of amino acids improve variables of nitrogen metabolism and productivity in soybean crop. Front Plant Sci. 2018;9:396. doi: 10.3389/fpls.2018.00396. 29643860 PMC5882785

[29] Marium A , Kausar A , Ashraf MY , Kanwal H , Nazli ZI . Enhancement in biomass and nutrient uptake in cucumber through methionine and phenylalanine under saline soils. Pak J Agric Sci. 2021;58(1):109–117. doi: 10.21162/PAKJAS/21.1034.

[30] Weber E , Bleiholder H . Erläuterungen zu den BBCH-Dezimal-Codes für die entwicklungsstadien von mais, raps, faba-bohne, sonnenblume und erbse – mit abbildungen. Gesunde Pflanz. 1990;42:308–321.

[31] Lancashire PD , Bleiholder H , Langelüddecke P , Stauss R , Van den Boom T , Weber E , Witzen-Berger A . A uniform decimal code for growth stages of crops and weeds. Ann Appl Biol. 1991;119(3):561–601. doi: 10.1111/j.1744-7348.1991.tb04895.x.

[32] Ahmad M , Waraich EA , Zulfiqar U , Hussain S , Yasin MU , Farooq M . Thiourea application improves growth, seed and oil yields in canola by modulating gas exchange, antioxidant defense, and osmoprotection under heat stress. J Soil Sci Plant Nutr. 2022;22(3):3655–3666. doi: 10.1007/s42729-022-00889-8.

[33] Arnon DI . Copper enzymes in isolated chloroplasts: polyphenol oxidase in *Beta vulgaris* . Plant Physiol. 1949;24(1):1–15. doi: 10.1104/pp.24.1.1. 16654194 PMC437905

[34] Velikova V , Yordanov I , Edreva A . Oxidative stress and some antioxidant systems in acid rain-treated bean plants: protective role of exogenous polyamines. Plant Sci. 2000;151(1):59–66. doi: 10.1016/S0168-9452(99)00197-1.

[35] Giannopolitis CN , Ries SK . Superoxide dismutases: I. Occurrence in higher plants. Plant Physiol. 1977;59(2):309–314. doi: 10.1104/pp.59.2.309. 16659839 PMC542387

[36] Chance B , Maehly AC . Assay of catalase and peroxidase. Methods Enzymol. 1955;2:764–775. doi: 10.1016/S0076-6879(55)02300-8.

[37] Marinova D , Ribarova F , Atanassova M . Total phenolics and flavonoids in Bulgarian fruits and vegetables. J Univ Chem Technol Metall. 2005;40(3):255–260.

[38] Murray JR , Hackett WP . Dihydroflavonol reductase activity in relation to differential anthocyanin accumulation in juvenile and mature phase *Hedera helix* L. Plant Physiol. 1991;97(1):343–351. doi: 10.1104/pp.97.1.343. 16668393 PMC1081004

[39] Mukherjee SP , Choudhuri MA . Implications of water stress-induced changes in the levels of endogenous ascorbic acid and hydrogen peroxide in vigna seedlings. Physiol Plant. 1983;58(2):166–170. doi: 10.1111/j.1399-3054.1983.tb04162.x.

[40] Kowalenko CG , Lowe LE . Determination of nitrates in soil extracts. Soil Sci Soc Am J. 1973;37(4):660–661. doi: 10.2136/sssaj1973.03615995003700040046x.

[41] Khorasaninejad S , Zare F , Hemmati K . Effects of silicon on some phytochemical traits of purple coneflower (*Echinacea purpurea* L.) under salinity. Sci Hortic. 2020;275:109684. doi: 10.1016/j.scienta.2020.109684.

[42] Saddiq MS , Iqbal S , Afzal I , Ibrahim AMH , Bakhtavar MA , Hafeez MB , Jahanzaib , Maqbool MM . Mitigation of salinity stress in wheat (*Triticum aestivum* L.) seedlings through physiological seed enhancements. J Plant Nutr. 2019;42(10):1192–1204. doi: 10.1080/01904167.2019.1609504.

[43] Cheng R , Zhu H , Cheng X , Shutes B , Yan B . Saline and alkaline tolerance of wetland plants: what are the most representative evaluation indicators? Sust. 2020;12(5):1913. doi: 10.3390/su12051913.

[44] Debez A , Chaibi W , Bouzid S . Effect of NaCl and growth regulators on germination of atriplex halimus L. Cah Agric. 2001;10:135–138.

[45] Pardo JM . Biotechnology of water and salinity stress tolerance. Curr Opin Biotechnol. 2010;21(2):185–196. doi: 10.1016/j.copbio.2010.02.005. 20189794

[46] Roy SJ , Negrão S , Tester M . Salt resistant crop plants. Curr Opin Biotechnol. 2014;26:115–124. doi: 10.1016/j.copbio.2013.12.004. 24679267

[47] Meng Y , Yin Q , Yan Z , Wang Y , Niu J , Zhang J , Fan K . Exogenous silicon enhanced salt resistance by maintaining K^+^/Na^+^ homeostasis and antioxidant performance in alfalfa leaves. Front Plant Sci. 2020;11:1183. doi: 10.3389/fpls.2020.01183. 32983188 PMC7479291

[48] Almeida DM , Oliveira MM , Saibo NJM . Regulation of Na^+^ and K^+^ homeostasis in plants: towards improved salt stress tolerance in crop plants. Genet Mol Biol. 2017;40(1 Suppl 1):326–345. doi: 10.1590/1678-4685-GMB-2016-0106. 28350038 PMC5452131

[49] Siddiqui H , Hayat S , Bajguz A . Regulation of photosynthesis by brassinosteroids in plants. Acta Physiol Plant. 2018;40(3):59. doi: 10.1007/s11738-018-2639-2.

[50] Chandrasekaran M , Chanratana M , Kim K , Seshadri S , Sa T . Impact of arbuscular mycorrhizal fungi on photosynthesis, water status, and gas exchange of plants under salt stress: a meta-analysis. Front Plant Sci. 2019;10:457. doi: 10.3389/fpls.2019.00457. 31040857 PMC6476944

[51] Abdalla A , Sadak MS , Abd Elhamid EM , Ezo M . Amelioration of drought stress reduced effects by exogenous application of L-phenylalanine on *Moringa oleifera* . Egypt J Chem. 2022;65(8):523–532. doi: 10.21608/EJCHEM.2022.113606.5208.

[52] Hashimoto H , Sugisaki M , Yoshizawa M . Ultrafast time-resolved vibrational spectroscopies of carotenoids in photosynthesis. Biochim Biophys Acta Bioenergy. 2015;1847(1):69–78. doi: 10.1016/j.bbabio.2014.09.009. 25223589

[53] Paul D , Lade H . Plant-growth-promoting rhizobacteria to improve crop growth in saline soils: a review. Agron Sustain Dev. 2014;34(4):737–752. doi: 10.1007/s13593-013-0153-2.

[54] Hoffmann J , Berni R , Hausman JF , Guerriero G . A review on the beneficial role of silicon against salinity in non-accumulator crops: tomato as a model. Biomolecules. 2020;10(9):1284. doi: 10.3390/biom10091284. 32906642 PMC7563371

[55] Azooz MM , Metwally A , Abou-Elhamd MF . Jasmonate-induced tolerance of hassawi okra seedlings to salinity in brackish water. Acta Physiol Plant. 2015;37:77. doi: 10.1007/s11738-015-1828-5.

[56] Atteya AK , El-Serafy RS , El-Zabalawy KM , Elhakem A , Genaidy EA . Exogenously supplemented proline and phenylalanine improve growth, productivity, and oil composition of salted moringa by up-regulating osmoprotectants and stimulating antioxidant machinery. Plants. 2022;11(12):1553. doi: 10.3390/plants11121553. 35736704 PMC9227737

[57] Butt M , Ayyub CM , Amjad M , Ahmad R . Proline application enhances growth of chili by improving physiological and biochemical attributes under salt stress. Pak J Agric Sci. 2016;53(1):43–49.

[58] Zhang Z , Mao C , Shi Z , Kou X . The amino acid metabolic and carbohydrate metabolic pathways play important roles during salt-stress response in tomato. Front Plant Sci. 2017;8:1231. doi: 10.3389/fpls.2017.01231. 28769946 PMC5511834

[59] Atteya AK , Albalwa AN , El-Serafy RS , Albalwa K , Bayomy HM , Genaidy EAE . Response of *Moringa oleifera* seeds and fixed oil production to vermicompost and NPK under calcareous soil conditions. Plants. 2021;10(10):1998. doi: 10.3390/plants10101998. 34685807 PMC8538915

[60] Semida WM , Abdelkhalik A , Rady MO , Marey RA , Abd El-Mageed TA . Exogenously applied proline enhances growth and productivity of drought-stressed onion by improving photosynthetic efficiency, water use efficiency, and up-regulating osmoprotectants. Sci Hortic. 2020;272:109580. doi: 10.1016/j.scienta.2020.109580.

[61] Berni R , Luyckx M , Xu X , Legay S , Sergeant K , Hausman JF , Lutts S , Cai G , Guerriero G . Reactive oxygen species and heavy metal stress in plants: impact on the cell wall and secondary metabolism. Environ Exp Bot. 2019;161:98–106. doi: 10.1016/j.envexpbot.2018.10.017.

[62] Koca N , Karaman S . The effects of plant growth regulators and L-phenylalanine on phenolic compounds of sweet basil. Food Chem. 2015;166:515–521. doi: 10.1016/j.foodchem.2014.06.100. 25053088

[63] Indra GS . Arulselvi P. Effect of cytokinin combined elicitors (L-phenylalanine, salicylic acid and chitosan) on in vitro propagation, secondary metabolites and molecular characterization of medicinal herb coleus aromaticus benth. J Saudi Soc Agric Sci. 2018;17(4):435–444. doi: 10.1016/j.jssas.2016.09.002.

[64] Shekari G , Javanmardi J . Effects of foliar application of pure amino acid and amino acid-containing fertilizer on broccoli (*Brassica oleracea* L. var. italica) transplants. Adv Crop Sci Technol. 2017;5(3):1–5. doi: 10.4172/2329-8863.1000280.

[65] Heldt HW , Piechulla B . Plant Biochemistry. 4th ed. Burlington, MA: Academic Press; 2010.

[66] Zali AG , Ehsanzadeh P . Exogenously applied proline as a tool to enhance water use efficiency: case of fennel. Agric Water Manag. 2018;197:138–146. doi: 10.1016/j.agwat.2017.11.008.

[67] Ninkuu V , Aluko OO , Yan J , Zeng H , Liu G , Zhao J , Li H , Chen S , Dakora FD . Phenylpropanoid metabolism: recent insights into stress tolerance and plant development cues. Front Plant Sci. 2025;16:1571825. doi: 10.3389/fpls.2025.1571825. 40641862 PMC12242330

[68] Pascual MB , El-Azaz J , de la Torre FN , Cañas RA , Avila C , Cánovas FM . Biosynthesis and metabolic fate of phenylalanine in conifers. Front Plant Sci. 2016;7:1030. doi: 10.3389/fpls.2016.01030. 27468292 PMC4942462

[69] Teixeira WF , Fagan EB , Soares LH , Umburanas RC , Reichardt K , Neto DD . Foliar and seed application of amino acids affects the antioxidant metabolism of the soybean crop. Front Plant Sci. 2017;8:327. doi: 10.3389/fpls.2017.00327. 28377778 PMC5359285

[70] Sadak MS , Abd El-Hameid ARA , Zaki FS , Dawood MG , El-Awadi ME . Physiological and biochemical responses of soybean (*Glycine max* L.) to cysteine application under sea salt stress. Bull Natl Res Cent. 2020;44(1):1–10. doi: 10.1186/s42269-020-00363-5.

[71] Stephens NR , Qi Z , Spalding EP . Glutamate receptor subtypes evidenced by differences in desensitization and dependence on the GLR3.3 and GLR3.4 genes. Plant Physiol. 2008;146(2):529–538. doi: 10.1104/pp.107.111450. 18162597 PMC2245834

[72] Zhang M , Zhai Z , Tian X , Duan L , Li Z . Brassinolide alleviated the adverse effect of water deficit on photosynthesis and antioxidant metabolism of soybean (*Glycine max* L.). Plant Growth Regul. 2008;56(3):257–264. doi: 10.1007/s10725-008-9305-4.

[73] Sadak MS , Abd Elhamid EM , Ahmed Mmrm L . Glutathione induced antioxidant protection against salinity stress in Chickpea (*Cicer arietinum* L.) plant. Egypt J Bot. 2017;57(2):293–302. doi: 10.21608/EJBO.2017.1078.1105.

[74] Abd-Allah EF , Hashem A , Alqarawi AA , Hend AA . Alleviation of adverse impact of cadmium stress in sunflower (*Helianthus annuus* L.) by arbuscular mycorrhizal fungi. Pak J Bot. 2015;47(2):785–795.

[75] El-Serafy RS , El-Sheshtawy AA , Atteya AK , Al-Hashimi A , Abbasi AM , Al-Ashkar I . Seed priming with silicon as a potential to increase salt stress tolerance in *Lathyrus odoratus* . Plants. 2021;10(10):2140. doi: 10.3390/plants10102140. 34685950 PMC8539537

[76] Al-Mushhin Aa . Interactive effect of potassium and spermidine protects growth, photosynthesis and chlorophyll biosynthesis in *Vigna angularis* from salinity-induced damage by up-regulating tolerance mechanisms. Not Bot Horti Agrobot Cluj Napoca. 2022;50(4):12607. doi: 10.15835/nbha50412607.

[77] Abd El-Samad HM , Shaddad MAK , Barakat N . The role of amino acids in improvement of salt tolerance of crop plants. J Stress Physiol Biochem. 2010;6(3):25–37 AbdBN.

[78] An M , Zhu Y , Chang D , Wang X , Wang K . Metabolic and photosynthesis analysis of compound-material-mediated saline and alkaline stress tolerance in cotton leaves. Plants. 2025;14(3):394. doi: 10.3390/plants14030394. 39942955 PMC11819938

[79] Rady MM , Taha RS , Mahdi AH . Proline enhances growth, productivity and anatomy of two varieties of *Lupinus termis* L. Grown under salt stress. S Afr J Bot. 2016;102:221–227. doi: 10.1016/j.sajb.2015.09.008.

[80] Henderson BC , Sanderson JM , Fowles A . A review of the foliar application of individual amino acids as biostimulants in plants. Discover Agriculture. 2025;3(1):69. 40376175 10.1007/s44279-025-00222-7 PMC12075294

